# The data of change in macrophage gene expression which induced by perilipin 1 overexpression

**DOI:** 10.1016/j.dib.2018.05.027

**Published:** 2018-05-15

**Authors:** Kohei Yamamoto, Hideaki Miyoshi, Kyu Yong Cho, Akinobu Nakamura, Andrew S. Greenberg, Tatsuya Atsumi

**Affiliations:** a Department of Rheumatology, Endocrinology and Nephrology, Faculty of Medicine, Graduate School of Medicine, Hokkaido University, Kita 15 Nishi 7, Kita-ku, Sapporo, Hokkaido 060-8638, Japan; b Jean Mayer USDA Human Nutrition Research Center on Aging, Tufts University, 711 Washington Street, Boston, MA 02111, USA

**Keywords:** PLIN1, perilipin 1, *Plin1Tg*, PLIN1 transgenic mice, *ApoeKO*, apolipoprotein E knockout mice, RT-PCR, reverse transcription polymerase chain reaction, perilipin1, PLIN1, Macrophage, Atherosclerosis

## Abstract

The data presented here are related to the research article entitled “Overexpression of Perilipin1 protects against atheroma progression in apolipoprotein E knockout mice” [1]. This paper describes data that were obtained from perilipin 1 (PLIN1) transgenic mice (*Plin1Tg*) regarding atherosclerosis. The main aim of collecting the data was to clarify the role of PLIN1 in the pathophysiology of atherosclerosis. The data were collected from C57BL/6J mice, apolipoprotein E knockout mice (*ApoeKO*) and *Plin1Tg/ApoeKO*. The atherosclerotic lesion areas of aorta were 3.3 ± 1.2% in C57BL/6J mice, 14.2 ± 3.2% in *ApoeKO*, and 5.6 ± 1.9% in *Plin1Tg/ApoeKO*. Body weight, gonadal adipose mass and plasma triglyceride concentrations were comparable among the three groups [1]. Furthermore, PLIN1 overexpression did not affect the gene expressions related to cholesterol influx and efflux in macrophage.

**Specifications Table**

**Table:** 

Subject area	*Medicine*
More specific subject area	*Atherosclerosis*
Type of data	*Table and text file*
How data was acquired	*Real-Time PCR (Applied Biosystems 7500 Fast Real-Time PCR System, Thermo Fisher)*
Data format	*Analyzed*
Experimental factors	*Data obtained from C57BL/6* *J mice, apolipoprotein E knockout mice and Plin1 transgenic mice*
Experimental features	*The effect of PLIN1 overexpression on atherosclerosis*
Data source location	*Hokkaido University, Sapporo, Japan*
Data accessibility	*The data are available with this article*

**Value of the data**

• Overexpression of PLIN1 in macrophages protected against atheroma progression.
• No major risk factors were altered in PLIN1 transgenic mice fed normal diet.
• Overexpression of PLIN1 did not affect the gene expressions related to cholesterol influx and efflux in macrophage

## 1 Data

Thioglycollate-elicited peritoneal macrophages were isolated from C57BL/6J mice or perilipin 1 (PLIN1) transgenic mice (*Plin1Tg*). Total RNA was prepared and analyzed by reverse transcription polymerase chain reaction (RT-PCR). Expression of the PLIN1 transgene and endogenous PLIN1 and PLIN2 genes in the *Plin1Tg* macrophages was confirmed. Body weight were 26.6 ± 3.1 g in C57BL/6J mice, 29.0 ± 4.5 g in apolipoprotein E knockout mice (*ApoeKO*), and 27.5 ± 3.9 g in *Plin1Tg/ApoeKO*. Gonadal fat mass were 356 ± 78 mg in C57BL/6J mice, 332 ± 124 mg in *ApoeKO*, and 424 ± 190 mg in *Plin1Tg/ApoeKO*. Plasma total cholesterol were 72 ± 11 mg/dl in C57BL/6J mice, 395 ± 80 mg/dl in ApoeKO, and 471 ± 138 mg/dl in Plin1Tg/ApoeKO. Plasma tumor necrosis factor-alpha were undetectable in all groups. Plasma interleukin-6 were 22 ± 14 pg/ml in C57BL/6J mice, 64 ± 37 pg/ml in *ApoeKO*, and 28 ± 31 pg/ml in *Plin1Tg/ApoeKO* [1].

The size of the atherosclerotic lesions were examined in the aortic sinus area and in the whole aorta using an en face method with Oil Red O staining. The lesions were quantified as a percentage of total aorta area. The atherosclerotic lesion areas of aorta were 3.3 ± 1.2% in C57BL/6 J mice, 14.2 ± 3.2% in ApoeKO, and 5.6 ± 1.9% in Plin1Tg/ApoeKO. [1].

**Fig. 1:**
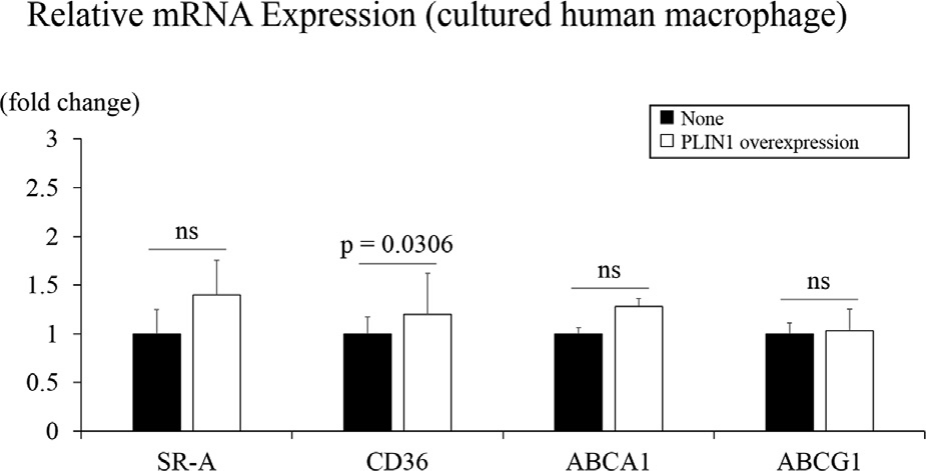
Relative mRNA Expression (cultured human macrophage).

Peritoneal thioglycollate-elicited macrophages were induced by acute inflammation and might be different in character from macrophages in plaques. We therefore used cultured human macrophages derived from monocytes to assess the expression of genes involved in cholesterol uptake and efflux. Although the CD36 expression level in PLIN1 overexpressed macrophages was 1.2 times higher than the control value, PLIN1 overexpression did not affect gene expression levels of SR-A, ABCA1 or ABCG1 (Fig. 1).

## 2 Experimental design, materials, and methods

### 2.1 Animal experiments

*Plin1Tg* were generated using the aP2 promotor on a C57BL/6J background as previously described. The PLIN1 expression level in white adipose tissue in *Plin1Tg* was shown to be two times higher than that in control mice [2]. *ApoeKO* were purchased from the Jackson Laboratory (Bar Harbor, United States). C57BL/6J mice were purchased from Charles River Japan (Yokohama, Japan). *Plin1Tg* and *ApoeKO* were crossed to obtain *Plin1Tg*/*ApoeKO* mice. The mice were housed at the Graduate School of Medicine's Institute for Animal Experimentation at Hokkaido University in accordance with the institutional guidelines of Hokkaido University Graduate School of Medicine. All mice were housed at room temperature, maintained on a 12 h light/dark cycle, and given free access to water.

All mice received a normal chow diet (MF from Oriental Yeast, Tokyo, Japan) for 20 weeks. Body weight and gonadal fat mass were measured. Blood was collected from inferior vena cava and plasma was obtained by centrifugation for enzymatic determination (Wako, Tokyo, Japan; R&D Systems, Minneapolis, United States) of lipid concentrations and proinflammatory cytokine levels. Aortic sinuses and whole aortas were collected for quantification of atheroma lesions. Data are expressed as means ± SD.

### 2.2 RT-PCR analysis

Thioglycollate-elicited macrophages were isolated from C57BL/6 J mice and *Plin1Tg* by washing the peritoneal cavity with 3 ml of phosphate-buffered saline one day after the mice were intraperitoneally injected with 50 μl of 4% thioglycollate in phosphate-buffered saline. Individual cell suspensions were washed with red blood cell lysis buffer (eBioscience, San Diego, United States). Total RNA was isolated from the isolated macrophages using an RNeasy Mini kit (QIAGEN, Venlo, Netherlands) according to the manufacturer's recommendations, and was used as the starting material for cDNA preparation. RT-PCR was performed using ReverTra-Plus (Toyobo, Osaka, Japan) in accordance with the manufacturer's protocols. Primer sequences are shown in Table 1.

**Table 1:** Primers used for detection of transgene expression.

	**Forward primer**	**Reverse primer**
*hPLIN1*	TCTCTCGATACACCGTGCAG	AGGGCTGCTACCTCACTGAA
*mPLIN1*	TGAAGGGTGTTACGGATAACG	ATGTCTCGGAATTCGCTCTC
*PLIN2*	GATTGAATTCGCCAGGAAGA	TGGCATGTAGTCTGGAGCTG
*GAPDH*	AACTTTGGCATTGTGGAAGG	ACACATTGGGGGTAGGAACA

### 2.3 Real-Time PCR analysis

Human monocytes were isolated from healthy control. Monocytes were incubated in RPMI 1640 (Thermo Fisher) with 10% pooled human serum at 37 degree 6 days, and derived to macrophage. Total RNA was isolated from the cultured macrophages using an RNeasy Mini kit (QIAGEN, Venlo, Netherlands) according to the manufacturer's recommendations. Real-Time PCR was performed using Applied Biosystems 7500 Fast Real-Time PCR System (Thermo Fisher) in accordance with the manufacturer's protocols. Data are expressed as means ± SD. Primer sequences are shown in Table 2.

**Table 2:** Primers used for Real-Time PCR.

	**Forward primer**	**Reverse primer**
*SR-A*	CCAGGGACATGGAATGCAA	CCAGTGGGACCTCGATCTCC
*CD36*	CTGTCATTGGTGCTGTCCTG	CTCAGCGTCCTGGGTTACAT
*ABCA1*	ACCTGCTGCCCTACAGTGAT	ATGGAGACCGAAGTGGTGAG
*ABCG1*	TGCAATCTTGTGCCATATTTGA	CCAGCCGACTGTTCTGATCA
